# Clean Energy and Health Expenditure in an African Setting: A Socioeconomic Analysis of Household Costs and Willingness to Pay

**DOI:** 10.36469/001c.158931

**Published:** 2026-03-20

**Authors:** Davies Adeloye, Wale O. Ogunleye, Boni M. Ale, Olayemi O. Akinnola, Alexander Iseolorunkanmi, Abimbola D. Akinyosoye, Emmanuel Okafor, Amos A. Olore, Akinyimika Sowunmi, Faatihah Niyi-Odumosu, Obianuju B. Ozoh, C2REST Nigeria Study

**Affiliations:** 1 Centre for Population Health and Healthcare, School of Health and Life Sciences, Teesside University, Middlesbrough, UK; 2 Department of Agricultural Economics, University of Ibadan, Ibadan, Nigeria; 3 University of Nairobi—Institute of Tropical and Infectious Diseases (UNITID), Nairobi, Kenya; 4 Holo Global Health Research Institute, Nairobi, Kenya; 5 Department of Biological Sciences, Covenant University, Ota, Nigeria; 6 Covenant University Medical Centre, Covenant University, Ota, Nigeria; 7 Covenant University; 8 Department of Medicine, Faculty of Clinical Sciences, College of Medicine, University of Lagos, Lagos, Nigeria; 9 Department of Sociology, Covenant University, Ota, Nigeria; 10 Faculty of Allied Health Sciences, Amadeus University, Amizi, Nigeria; 11 School of Applied Sciences, University of the West of England, Bristol, UK

**Keywords:** respiratory health, household expenditure, climate change, clean energy, Nigeria

## Abstract

**Background:**

Climate vulnerability in sub-Saharan Africa, including Nigeria, has heightened concern about household energy transitions and associated health and economic impacts. Despite clean fuel initiatives, uncertainty remains regarding household expenditure patterns, health expenditures, and socioeconomic drivers of adoption.

**Objectives:**

To examine (1) household cooking-energy and respiratory healthcare expenditures, (2) descriptive expenditure ratios comparing energy costs with respiratory healthcare spending including benefits of transitioning to cleaner energy solutions, and (3) the socioeconomic determinants that influence households’ willingness to transition to cleaner cooking practices.

**Methods:**

A cross-sectional household survey was conducted in Alimosho (Lagos State) and Ado-Odo Ota (Ogun State) in southwest Nigeria. Using a multistage cluster sampling approach, 292 respondents from 200 households were surveyed on cooking-energy expenditures, respiratory healthcare costs, and willingness to adopt clean fuels. Expenditure patterns were compared descriptively, and a model-based cost-utility analysis was performed to estimate the incremental cost-effectiveness ratio in US dollars per disability-adjusted life-years (DALYs) averted. A multivariable logistic regression model was used to examine socioeconomic predictors of willingness to adopt clean cooking.

**Results:**

Clean-fuel (liquefied petroleum gas or electricity) households reported higher annual cooking-energy expenditures than polluting-fuel households (US25.08vsUS16.27), as well as higher respiratory healthcare expenditures (US112.50vsUS50.64). The healthcare-to-energy expenditure ratio was also higher among clean-fuel users (4.48 vs 3.11). In adjusted analyses, tertiary education was associated with higher willingness to adopt clean cooking, while larger household size and urban residence were associated with lower willingness. In the model-based economic evaluation over a 1-year horizon, clean cooking was cost-saving (dominant) across plausible DALY-averted scenarios.

**Conclusions:**

Expenditure differences by fuel type likely reflect underlying socioeconomic conditions and variations in healthcare access, rather than causal effects of fuel choice. The model-based cost-utility analysis suggests that clean cooking could be cost-saving or highly cost-effective under plausible assumptions. Policies that address affordability and supply constraints, alongside stronger longitudinal evidence, are needed to support equitable and sustained clean-cooking transitions in Nigeria and across sub-Saharan Africa.

## BACKGROUND

Africa faces a disproportionate share of climate change impacts and health risks, despite contributing minimally to global greenhouse gas emissions.^1^ In sub-Saharan Africa (SSA), climate-related losses, including health impacts, have increased from an estimated US$7 billion to US$15 billion annually since 2020, with projections reaching up to US$50 billion by 2030.^2^ Among other health challenges, the increasing health burden, including respiratory diseases, driven partly by rising temperatures, extreme weather events, and deteriorating air quality, has emerged as a major concern.^3^

Across SSA, over 70% of households depend on traditional biomass fuels, such as wood and charcoal, for domestic cooking.^4,5^ This reliance increases risk of chronic respiratory diseases, including asthma, chronic obstructive pulmonary disease, and lung cancer, with vulnerable groups like women, children, and the elderly most affected.^6,7^ Despite clean energy alternatives, including liquefied petroleum gas (LPG) and improved biomass stoves, demonstrating some promise in mitigating pollution-related health risks, many households in these settings are still without these options.^8–10^ The household energy challenges observed in Nigeria mirror those across much of SSA, where more than 80% of the population still relies on solid fuels for cooking. Regional evidence from Ghana, Kenya, Malawi, and Uganda highlights that similar combinations of affordability constraints, unreliable infrastructure, and entrenched cooking practices slow the uptake of clean energy technologies.^11,12^ The CLEAN-Air (Africa) project, for example, underscores the substantial health benefits that cleaner cooking using LPG can deliver in countries such as Cameroon, Kenya, Tanzania, Rwanda, and Uganda.^11,12^

In Nigeria, the health impacts of climate change compound pre-existing population vulnerabilities stemming from poverty, poor housing, and inadequate living conditions, with the country now ranked 27th worldwide in climate-related fatalities.^13^ Besides, economic losses from these adverse climate events further strain a public health system already challenged by other climate-related health risks, including respiratory diseases.^14^ Although initiatives such as the Nigeria Distributed Access through Renewable Energy Scale-up (DARES) and the Nigeria Electrification Project (NEP) have broadened access to electricity, millions of Nigerians, particularly those living in rapidly growing slums and informal settlements which now characterize many urban settings, continue to rely on biomass fuels and polluting options for household energy.^15–17^ Reliance on polluting fuels not only exacerbates greenhouse gas emissions but also contributes to poor indoor air quality and harsh household thermal conditions.^18^

Socioeconomic and institutional factors, including education, income, urban-rural disparities, and infrastructural development, significantly shape household energy choices and, by extension, the broader health implications.^19–21^ Consequently, as households persist in using polluting energy sources, they inadvertently continue to reinforce climate change. We therefore examined (1) household cooking-energy and respiratory healthcare expenditures, (2) descriptive expenditure ratios comparing energy costs with respiratory healthcare spending including benefits of transitioning to cleaner energy solutions, and (3) the socioeconomic determinants that influence households’ willingness to transition to cleaner cooking practices.

## METHODS

### Study Design

We conducted a cross-sectional household survey in Alimosho (Lagos State) and Ado-Odo Ota (Ogun State), southwest Nigeria. The household head (or up to two responsible adults where applicable) was interviewed on household cooking energy expenditures, respiratory health-related costs, and willingness to transition to clean cooking. A total of 200 households were targeted (100 per site), yielding 292 completed respondent interviews. The study was conducted between 1 June 2024 and 30 November 2024, in line with the STROBE guidelines (see **Supplementary Material**).^22^

### Study Area

This study was conducted in two Nigerian local government areas: Alimosho in Lagos State and Ado-Odo Ota in Ogun State. These locations were purposefully selected due to their shared border and complementary characteristics, which make both ideal for examining climate risks and clean energy utilization within rapidly urbanizing contexts in Nigeria and broader SSA. Alimosho has the largest population in Lagos, Africa’s most populous city, with an estimated population of over 21 million in 2025. Ado-Odo/Ota is the most industrialized part of Ogun State and Nigeria, and it represents an important peri-urban industrial hub for investigating climate-related challenges and sustainable energy practices. Both study sites typify the rapidly urbanizing, mixed-energy economies that characterize many African cities, facilitating comparison of findings and future replication across diverse SSA contexts.

### Data Collection

Data for this study were collected using a structured questionnaire (see **Supplementary Material**). The questionnaire was adapted from prior validated household energy and health surveys in African settings to ensure relevance and reliability.^23,24^ The instrument contained sections on sociodemographic characteristics, energy sources and costs, health expenditures, and willingness to pay for cleaner energy. Before full-scale implementation, the questionnaire was piloted on a small sample of households similar to the main study population. Trained enumerators subsequently administered the final version through face-to-face interviews. Data were captured electronically using KoBoToolbox (Harvard Humanitarian Initiative, Cambridge, Massachusetts), which enabled real-time consistency checks and efficient data management.

### Case Definitions

For this study, we worked with the following case definitions:

- **Climate-related health risks:** These refer primarily to respiratory diseases or worsening respiratory symptoms exacerbated by environmental changes such as extreme heat, poor air quality, and increased pollution.^25^
- **Clean energy:** In this study, “clean energy” refers to LPG and electricity, while “polluting fuels” include firewood, charcoal, and kerosene. We avoided the term “fossil fuels” for these traditional fuels, as they are more accurately classified as biomass or kerosene-based polluting fuels.^15^
- **Household expenditure:** This refers to the monetary outlays on health-related needs, particularly expenditures on respiratory healthcare services or interventions within the Nigerian context.^20,21^ We summarized expenditure patterns by comparing the costs of adopting LPG-based clean cooking solutions with self-reported respiratory healthcare expenditures.
- **Willingness to pay:** Willingness to pay (WTP) is the maximum amount that households are prepared to spend on clean cooking solutions, specifically LPG, to mitigate climate-related respiratory risks.^19^
- **Cost-effectiveness:** Cost-effectiveness evaluates the balance between the costs of adopting LPG-based clean cooking solutions and the resulting reductions in respiratory disease burden in Nigeria.^14^

### Participants

**Sampling technique:** A multistage cluster random sampling method was employed to select the study population. First, communities within each local government area were randomly selected from comprehensive lists provided by the local authorities, with careful consideration given to the industrial and transport dynamics that influence exposure to climate risks. In the next stage, detailed maps obtained from local government offices were used to identify two distinct communities within each selected area. Within these communities, households were randomly sampled, and the household head (or up to two responsible adults when the household head was unavailable) was interviewed.

**Sample size estimation**: The C2REST Nigeria Study primarily explored the impacts of climate change on respiratory health. Hence, based on a prevalence of current wheeze and chronic obstructive pulmonary disease of 9% in Nigeria and an assumed relative risk of 2,^26^ a 2-sided significance level of 5% and a power of 80% was adopted for the calculation, an initial estimate of 452 participants. Considering that the average household size in Nigeria in 2019 was approximately 5.06 people, the adjusted sample size corresponded to an estimated 98 households, which was rounded up to 100 households per study location.^27,28^

**Recruitment:** Prior to participant recruitment, local community leaders were engaged to facilitate entry into the communities and ensure that the study was conducted with the community’s involvement and support.

### Statistical Analyses

We compared household expenditures associated with cooking-energy use and respiratory healthcare. Current household expenditures were assessed using self-reported annual cooking-energy costs and respiratory healthcare spending. These were summarized descriptively and explored as cross-sectional associations with primary fuel type. The observed costs were then compared with potential savings or avoided expenses anticipated from the adoption of clean energy interventions, reflected through reduced healthcare costs and improved productivity. Several contextual assumptions were applied to standardize comparisons across households, including average household size, income levels, and climate-related vulnerability, based on the Nigerian context.^27,28^

**Healthcare-to-energy expenditure ratio:** We estimated a healthcare–energy expenditure ratio (HER) to assess the relative health cost burden associated with clean vs polluting fuels. The HER was defined as the ratio of annual healthcare expenditures to annual cooking energy expenditures:

For clean-energy households:

$$HER_{clean}=\frac{\text{Annual healthcare cost}}{\text{Annual clean energy cost}}$$

For polluting-fuel households:

$$HER_{polluting}=\frac{\text{Annual healthcare cost}}{\text{Annual polluting fuel cost}}$$

Clean-energy costs included annual expenditures on LPG and electricity. Polluting-fuel costs included annual spending on firewood, charcoal, and kerosene. Healthcare costs encompassed self-reported expenditures related to respiratory and associated conditions, including cough, bronchitis, pneumonia, eye irritation or infection, skin conditions, stroke, cataract, and lung cancer.

A multivariable logistic regression model was used to examine socioeconomic and demographic factors influencing willingness to adopt cleaner energy. The dependent variable was binary, indicating whether a household was willing to change to clean energy (1) or not (0) and the independent variables were age and sex of the household head (both significant in the bivariate analysis). The model is specified as:

$$\ln\left(\frac{P_{i}}{1-P_{i}}\right)=\beta_{0}+\beta_{1}X_{1}+\beta_{2}X_{2}+\cdots +\beta_{n}X_{n}+\varepsilon$$

where *P_i_* is the probability that a household is willing to adopt clean energy, and 1 − Pi is the probability of not being willing. *β_0_…β_n_* are the coefficients to be estimated, *X_1_…X_n_* are the independent variables and ε is the error term.

**Cost-utility analysis:** We conducted a model-based cost-utility analysis comparing households using clean-only cooking fuels (LPG or electricity) with those using any polluting fuels, including fuel stacking. The analysis was conducted from the household perspective using a 1-year time horizon.

Annual household cooking costs were defined as the sum of annual fuel expenditures and annualized capital costs of stoves and cylinders. Capital costs were annualized assuming a 5-year lifespan. All costs were converted from Nigerian naira to US dollars using a period-average exchange rate. DALY reductions were estimated using two approaches:

1. **Base-case analysis**: A burden-share model derived from national household air-pollution-attributable DALY estimates, adjusted for household size and assumed adoption effectiveness.
2. **Sensitivity analyses**: Exposure-response scenarios assuming DALY reductions of 0.03 to 0.10 per household-year.

Incremental cost-effectiveness ratios (ICERs) were calculated as the incremental cost per DALY averted. Cost-effectiveness was evaluated using a willingness-to-pay threshold equivalent to Nigeria’s gross domestic product (GDP) per capita (US$2160 per DALY averted). Deterministic and probabilistic sensitivity analyses were conducted to assess uncertainty. Exploratory simulation analyses were conducted as a hypothesis-generating sensitivity exercise to illustrate potential patterns under larger hypothetical samples. Given their assumption-driven nature, these results are not presented as empirical evidence and are provided in the **Supplementary Material**.

### Ethics

Formal written informed consent was secured from the heads of the selected households and from every individual member prior to enrollment in the study. The Health Research Ethics Committee of Lagos University Teaching Hospital (HREC: 19/12/2008A) and Covenant University (CU/HREC/333/24) granted ethical clearance. Additionally, the study received social approval and clearance from the Lagos State Government (LSMH/4686/1/27) and the Ogun State Government (CHREC/467/25/APP).

## RESULTS

### Study Participants and Cooking Choices

A total of 292 households from 200 households were included in the analysis, of which 239 (82%) used clean cooking fuels exclusively, and 53 (18%) used polluting fuels or stacked fuels. The mean age of household heads was 46.0 years (standard deviation [SD] = 13.4). Household heads using polluting fuels were significantly older than those using clean fuels (50.1 vs 45.0 years, *P* = .019). Most household heads were male (84%), although a higher proportion of female-headed households was observed among polluting-fuel users compared with clean-fuel users (25% vs 15%, *P* = .079). Urban households accounted for the majority (58%), but a higher proportion of clean-fuel users lived in rural or peri-urban areas than polluting-fuel users (45% vs 26%, *P* = .012).

Marital status differed across groups (*P* = .028), with single household heads more commonly represented among clean-fuel users (18% vs 8%). Educational attainment was also significantly associated with cooking fuel choice (*P* = .008). Among clean-fuel households, 45% of household heads had tertiary education, compared with 23% among polluting-fuel households. Occupational status did not differ significantly between groups (*P* = .079), although informal or self-employment was the most common form of work across both groups (66% overall). Household size was significantly larger among polluting-fuel users (mean, 5.5; SD = 2.3) compared with clean-fuel users (mean, 4.4; SD  =  2.1; *P* = .001) (**Table 1**).

**Table 1.** Study Participants’ Characteristics Stratified by Cooking Choices

**Characteristic**	**Overall (N = 292)**	**Polluting Fuels (n = 53)**	**Clean-⁠Only Fuels (n = 239)**	***P***
**Demographic characteristics**				
Age of household head, mean (SD)	46.0 (13.4)	50.1 (14.3)	45.0 (13.0)	.019
Sex of household head, n (%)				.079
Male	244 (83.6)	40 (75.5)	204 (85.4)	
Female	48 (16.4)	13 (24.5)	35 (14.6)	
Location, n (%)				
Rural	122 (41.8)	14 (26.4)	108 (45.2)	.012
Urban	170 (58.2)	39 (73.6)	131 (54.8)	
Marital status, n (%)				
Single	48 (16.4)	4 (7.5)	44 (18.4)	.028
Married	215 (73.6)	39 (73.6)	176 (73.6)	
Divorced/separated	10 (3.4)	3 (5.7)	7 (2.9)	
Widow/widower	19 (6.5)	7 (13.2)	12 (5.0)	
**Socioeconomic characteristics**				
Education level, n (%)				
No formal	5 (1.7)	2 (3.8)	3 (1.3)	.008
Primary	39 (13.4)	11 (20.8)	28 (11.7)	
Secondary	129 (44.2)	28 (52.8)	101 (42.3)	
Tertiary	119 (40.8)	12 (22.6)	107 (44.8)	
Main occupation, n (%)				.079
Unemployed/retired	12 (4.1)	2 (3.8)	10 (4.2)	
Agriculture	6 (2.1)	2 (3.8)	4 (1.7)	
Informal/self-employed	194 (66.4)	41 (77.4)	153 (64.0)	
Formal employment	80 (27.4)	8 (15.1)	72 (30.1)	
**Household characteristics**				
Household size, mean (SD)	4.6 (2.2)	5.5 (2.3)	4.4 (2.1)	<.001

Abbreviation: SD, standard deviation.

### Household Expenditure on Cooking Energy and Respiratory Health

Clean-fuel households reported higher annual cooking-energy expenditures than polluting-fuel households (US$25.08 [₦37 510] vs US$16.27 [₦24 271]). Similarly, mean annual respiratory healthcare expenditures were higher among clean-fuel households than among polluting-fuel households (US$112.50 [₦167 911] vs US$50.64 [₦75 554]). These differences likely reflect underlying socioeconomic status, healthcare access, and health-seeking behavior rather than effects attributable to fuel choice, given the cross-sectional study design.

**Figure 1.**
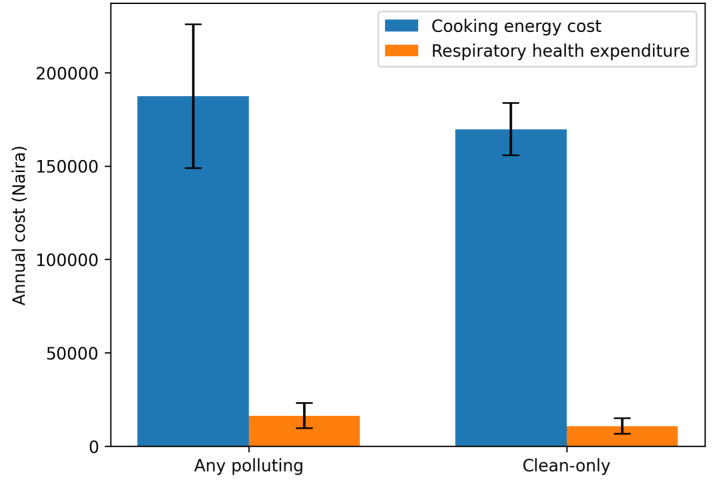
Mean Annual Cooking Energy and Respiratory Healthcare Expenditures by Fuel Type Note: Currency conversions use a period-average exchange rate of ₦1475 per US$1.

**Figure 2.**
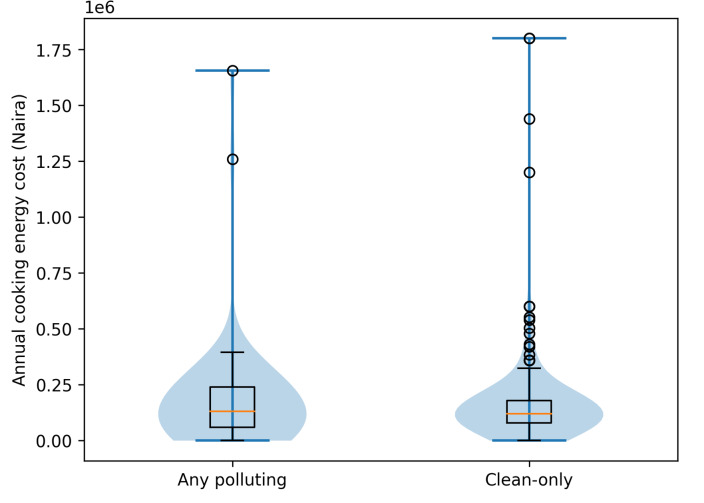
Distribution of Annual Cooking Energy Costs by Fuel Type The violin plot illustrates the density of annual cooking energy expenditures, while the overlaid box plot shows the median and interquartile range for households using polluting fuels and those using clean-only fuels.

**Figure 1** presents the mean annual cooking-energy and respiratory healthcare expenditures by fuel type. Clean-fuel households showed higher median expenditures and a wider interquartile range, indicating greater variability in spending patterns. The kernel density plots (**Figure 2**) suggest that some clean-fuel households incur substantially higher cooking-energy costs, while polluting-fuel households display a more compressed cost distribution.

The healthcare-energy expenditure ratio was higher among clean-fuel households (4.48) than among polluting-fuel households (3.11) (**Table 2**).

**Table 2.** Household Expenditures and Model-Based Cost-Utility Results by Fuel Type

**Measure**	**Polluting Fuels**	**Clean-⁠Only Fuels**	**Increment (Clean **−** Polluting)**	**ICER Result**
Observed annual household expenditures				
Cooking energy, ₦ (US$)	24 271 (16.27)	37 510 (25.08)	+13 239 (+8.81)	–
Respiratory healthcare, ₦ (US$)	75 554 (50.64)	167 911 (112.50)	+92 357 (+61.86)	–
Healthcare-energy ratio	3.11	4.48	+1.37	–
Model-based cost-utility results				
Annual cooking cost, ₦	184 000	122 000	−62 000	–
Annual cooking cost, US$	125	83	−42	–
DALYs averted per household-year				
Conservative scenario	–	–	0.03	Dominant (cost-⁠saving)
Base-case scenario	–	–	0.05	Dominant (cost-⁠saving)
Optimistic scenario	–	–	0.10	Dominant (cost-⁠saving)

Note: Observed expenditures represent mean annual household values. Model-based cooking costs include fuel plus annualized stove and cylinder costs (5-year lifespan). Currency conversion used ₦1475 per US$1. DALYs averted were model-based (0.03-0.10 per household-year). ICER = incremental cost per DALY averted; “dominant” indicates lower cost and greater effectiveness relative to polluting fuels. Cost-effectiveness was assessed at thresholds of US$500, US$1000, and US$2160 per DALY.

### Cost Utility

**Figure 3.**
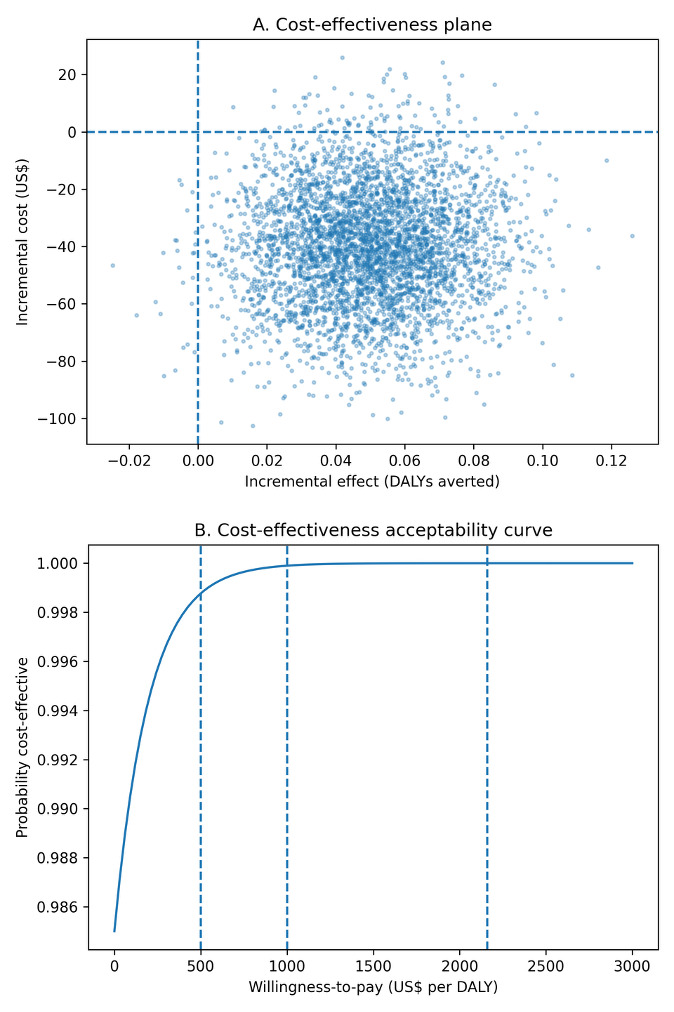
Probabilistic Cost-Effectiveness Results for Clean Cooking Panel A shows probabilistic simulations of incremental cost and effect for clean cooking vs polluting fuels; points in the southeast quadrant indicate cost-saving and greater effectiveness. Panel B shows the probability that clean cooking is cost-effective across willingness-to-pay thresholds; the probability exceeds ~99% at US$500 and US$1000 per DALY and remains high at the GDP-per-capita reference threshold (US$2160).

From our model-based cost-utility analysis, however, we observed that annual household cooking costs were lower among clean-only households (₦122 000; US$83) compared with households using any polluting fuels (₦184 000; US$125), yielding an incremental annual saving of approximately ₦62 000 (US$42) per household. Using the burden-share DALY model, clean cooking was estimated to avert approximately 0.05 DALYs per household per year in the base-case scenario. Unlike the observed survey expenditures, the model-based analysis incorporates standardized fuel use, stove capital costs, and avoided health expenditures, explaining the lower cost estimates for clean cooking. Under these assumptions, clean cooking was both less costly and more effective than polluting fuels, and therefore economically dominant (**Figure 3**). Across conservative and optimistic health-gain scenarios (0.03-0.10 DALYs averted per household-year), clean cooking remained cost-saving. The intervention remained highly cost-effective across conservative willingness-to-pay thresholds of US$500 and US$1000 per DALY averted, and at the GDP-per-capita reference threshold for Nigeria (US$2160 per DALY averted). Probabilistic sensitivity analysis (10 000 iterations) indicated a 71% probability that clean cooking was cost-saving and a 97% probability that it was cost-effective at the GDP-per-capita threshold (**Table 2**, **Figure 3**).

### Factors Associated with Willingness to Adopt Clean Cooking

**Table 2** presents the socioeconomic and demographic factors that influence households’ willingness to adopt clean cooking energy. Household size was a significant factor, with each additional household member reducing the likelihood of willingness to switch to clean energy by approximately 20% (adjusted odds ratio [AOR], 0.8; 95% confidence interval [CI], 0.7-0.9; *P* = .006). The education level of the household head also played an important role. Compared with households headed by individuals with only primary education, households headed by individuals with tertiary education were 2.7 times more likely to express a willingness to adopt clean energy (AOR, 2.7; 95% CI, 1.1-7.2; *P* = .033), whereas households headed by individuals with secondary education showed no significant difference. Location was a significant determinant, with households in urban areas 60% less likely to be willing to use clean cooking energy than those in rural areas (AOR, 0.4; 95% CI, 0.2-0.8; *P* = .012). Other variables, such as sex, age, marital status, and the household head’s employment status, were not significantly associated with willingness to adopt clean cooking energy in the adjusted model (**Table 3**).

**Table 3.** Factors Associated with Willingness to Use Clean Energy

**Variable**	**Category**	**Yes**	**No**	**Unadjusted OR (95% CI)**	***P***	**Adjusted OR (95% CI)**	***P***
Age (years)	Continuous	–	–	0.97 (0.94–0.99)	.013	0.99 (0.96–1.02)	.615
Sex	Male	204	40	Reference		Reference	
	Female	35	13	0.5 (0.2–1.1)	.082	0.5 (0.2–1.2)	.138
Location	Rural	108	14	Reference		Reference	
	Urban	131	39	0.4 (0.2–0.8)	.013	0.4 (0.2–0.8)	.012
Marital status	Not married	63	14	Reference		–	–
	Married	176	39	1.0 (0.4–1.9)	.993	–	–
Education	Primary	31	13	Reference		Reference	
	Secondary	101	28	1.5 (0.6–3.2)	.292	1.0 (0.4–2.3)	.948
	Tertiary	107	12	3.7 (1.5–9.1)	.003	2.7 (1.1–7.2)	.033
Occupation	Unemployed	10	2	Reference		–	–
	Employed	229	51	0.9 (0.1–3.5)	.891	–	–
Household size	Continuous	–	–	0.8 (0.7–0.9)	.002	0.8 (0.7–0.9)	.006

Abbreviations: CI, confidence interval; OR, odds ratio.

## DISCUSSION

This cross-sectional study provides household-level evidence on cooking fuel choice, expenditures on cooking energy and respiratory healthcare, and willingness to adopt clean cooking in two southwest Nigerian settings. We found that clean-fuel households reported higher cooking-energy expenditures and higher respiratory healthcare expenditures than households using polluting fuels. Given the cross-sectional design and likely socioeconomic confounding, these differences should be interpreted as associations that may reflect healthcare access and health-seeking behavior rather than effects attributable directly to fuel choice. Similar patterns have been reported elsewhere, where more affluent or educated households are both more likely to adopt clean fuels and to utilize formal healthcare services, resulting in higher reported expenditures despite potentially lower disease burden.^29,30^

The higher cooking-energy expenditures among clean-fuel households also likely reflect the market price and supply dynamics of LPG and electricity relative to polluting fuels, many of which are collected at little or no direct monetary cost. This aligns with global and regional evidence showing that affordability remains a central barrier to sustained adoption of clean cooking technologies.^12,31,32^ Higher reported respiratory healthcare spending among clean-fuel households may similarly reflect differences in health awareness, care-seeking behavior, and ability to pay. Globally, household air pollution remains a leading environmental risk factor, contributing substantially to respiratory and cardiovascular disease burden, particularly in low- and middle-income countries.^33,34^ These patterns underscore the need to address affordability and access barriers alongside behavioral and informational determinants of clean-cooking adoption.

To complement the descriptive expenditure findings and to explore the longer-term economic implications of fuel transitions, we conducted a model-based cost-utility analysis. Under base-case assumptions, clean cooking was both less costly and more effective than polluting fuels, and therefore economically dominant. Even under conservative assumptions about health gains, the intervention remained highly cost-effective at thresholds commonly used in low- and middle-income countries. These findings are consistent with earlier economic evaluations showing that clean-cooking interventions can yield substantial net benefits when reductions in healthcare costs, time burdens, and environmental impacts are considered.^35,36^ However, these estimates were derived from modeled parameters and should be interpreted as policy-relevant scenarios rather than causal effects derived directly from the survey. This apparent contrast reflects the different nature of the analyses, as the model incorporates standardized fuel use, stove capital costs, and avoided health expenditures. The survey captures observed spending patterns shaped by socioeconomic status and healthcare access, whereas the model estimates the expected costs and health gains attributable to fuel choice under standardized assumptions (see **Supplementary Material**).

In adjusted analyses, tertiary education of the household head was associated with a higher willingness to adopt clean cooking, consistent with evidence that education improves risk awareness and the capacity to act on health information.^29,31,37^ Household size was inversely associated with willingness to adopt clean cooking, suggesting that larger households may face greater affordability constraints due to higher fuel demand. Similar relationships have been observed across SSA, where larger households often rely on polluting fuels or engage in fuel stacking as a coping strategy.^31,38^

Urban households in our study were less likely to report willingness to adopt clean cooking than rural or peri-urban households. Although urban areas often have better physical access to modern fuels, affordability pressures, insecure housing, and price volatility may constrain adoption. Fuel stacking is common in urban African settings and may partly explain these findings.^30,38^ These socioeconomic gradients suggest that policies to accelerate clean-cooking transitions should prioritize affordability, such as targeted subsidies, microfinance, or pay-as-you-go models, alongside reliable supply chains and tailored behavior-change communication. Particular attention may be needed for larger households and groups with lower educational attainment. Strengthening access pathways in urban settings, where willingness may be constrained despite proximity to markets, could be especially important.

Although our data were drawn from two Nigerian settings, the economic and behavioral determinants identified here mirror structural patterns reported across SSA. Studies from Ghana, Kenya, and Ethiopia have documented similar socioeconomic gradients in fuel choice and adoption behavior.^10,39,40^ At a broader scale, such household-level decisions aggregate into macro-level energy demand patterns, with implications for electrification planning, climate mitigation, and public health outcomes across the region.^34,41^

This study has several strengths, including the use of primary household-level data, detailed measures of cooking-energy and healthcare expenditures, and the joint analysis of fuel use patterns and willingness to adopt clean cooking.

However, several limitations should be considered. First, the cross-sectional design precludes causal inference. Observed expenditure differences are likely influenced by underlying socioeconomic factors, healthcare access, and health-seeking behavior, rather than fuel effects alone. Second, expenditure data were self-reported and may be subject to recall bias. Healthcare expenditures were not clinically verified and may reflect utilization patterns rather than true differences in disease burden.

Third, fuel stacking was common, and the duration or consistency of clean-fuel use was not captured. This limited our ability to assess sustained exposure reductions or long-term health and economic effects. The absence of linked clinical or hospital data also restricted our ability to validate reported health expenditures or directly associate fuel use with specific medical diagnoses. Fourth, the healthcare-energy expenditure ratio was used as a descriptive indicator rather than a standard economic outcome. To address this, we conducted a model-based cost-utility analysis using externally derived household air-pollution burden parameters. These results represent scenario-based estimates and are sensitive to modeling assumptions rather than causal effects derived from the survey data.

Fifth, the logistic regression model assumes a linear relationship between predictors and the log-odds of willingness to adopt clean energy, which may not fully capture the complex socioeconomic, cultural, and infrastructural drivers of household energy decisions. The exploratory simulation analyses were also assumption-driven and should be interpreted as hypothesis-generating rather than confirmatory. Future research should use longitudinal or quasi-experimental designs to examine the causal health and economic impacts of transitions to clean cooking. Integrating clinical outcomes, routine health record data, and objective exposure measures would strengthen health impact assessment and provide more robust evidence for policy and investment decisions.

Despite these limitations, this study provides novel household-level evidence from Nigeria, combining empirical expenditure data with model-based economic evaluation to explore the potential health and economic implications of clean cooking transitions.

### Implications

One important implication relates to the need for stronger causal evidence. To provide stronger evidence on causal long-term effects, future research should employ longitudinal cohort designs or quasi-experimental approaches that can credibly capture changes in health outcomes following clean fuel adoption. Such designs would more directly address whether higher energy expenditures are offset by reductions in healthcare costs over time. The findings underscore a clear opportunity for integrating cost-effective clean energy interventions into national climate and health policies. Despite higher upfront energy costs, the long-term benefits, such as reduced healthcare expenditures, make a strong case for prioritizing these solutions. Practical implementation should focus on a few key interventions that have proven effective, such as transitioning households to clean cooking solutions like LPG or electricity.^41,42^ Other interventions that may be considered include improving ventilation within homes, using green building materials to reduce indoor air pollution, and promoting community tree-planting initiatives to create natural air quality buffers.^43,44^

However, several barriers could impede these efforts. Cultural preferences for traditional cooking methods, financial constraints, and insufficient infrastructure, especially in rural areas, present significant challenges. To overcome these hurdles, a coordinated approach is needed. Governments should consider introducing targeted subsidies and microfinancing options to ease the financial burden on households, while simultaneously investing in the necessary infrastructure to support widespread clean energy adoption. Nongovernmental organizations can play a crucial role by running educational campaigns that highlight the long-term health and economic benefits of clean energy, thereby increasing community acceptance. The private sector, in turn, should be encouraged to invest in innovative clean energy technologies and collaborate with public agencies to extend these solutions to underserved regions.

A practical path forward also involves establishing clear monitoring and evaluation metrics to track progress. For example, policymakers could measure the reduction in healthcare costs associated with respiratory diseases, the number of households switching to clean energy, and changes in the energy-to-healthcare cost ratios over time. Other useful indicators might include improvements in overall quality of life and socioeconomic markers such as increased household income and reduced disparities between urban and rural areas.

Extending the analysis beyond Nigeria, a coordinated regional approach across SSA could leverage shared policy frameworks, such as the African Union’s Agenda 2063 and the ECOWAS Regional Centre for Renewable Energy and Energy Efficiency, to scale up clean-energy adoption and financing.^45^ Cross-border collaborations could harmonize subsidy schemes, enhance LPG distribution networks, and strengthen data systems that track health and economic outcomes of energy transitions.^46^ Moreover, embedding behavioral insights and community participation within energy policy design can ensure that clean-energy interventions align with everyday practices, gender roles, and household economies across diverse African contexts.

## CONCLUSIONS

In these southwest Nigerian communities, clean-fuel households reported higher cooking-energy and respiratory healthcare expenditures than polluting-fuel households. These patterns likely reflect underlying socioeconomic differences and variations in healthcare access, rather than causal effects of fuel choice. Education, household size, and location emerged as key determinants of willingness to adopt clean cooking. Complementary model-based cost-utility analysis suggests that clean cooking could be potentially cost-saving or highly cost-effective under a range of plausible assumptions. Together, these findings highlight the need for policies that address affordability, supply reliability, and equitable access to clean fuels. Strengthening longitudinal and clinically linked evidence will also be important to support sustained clean-cooking transitions in Nigeria and across SSA.

### Disclosures

The authors have no conflicts of interest to disclose.

### Ethics Statement

Formal written informed consent was secured from the heads of the selected households and from every individual member prior to enrollment in the study. The Health Research Ethics Committee of Lagos University Teaching Hospital (HREC: 19/12/2008A) and Covenant University (CU/HREC/333/24) granted ethical clearance. Additionally, the study received social approval and clearance from the Lagos State Government (LSMH/4686/1/27) and the Ogun State Government (CHREC/467/25/APP).

## Supplementary Material

Online Supplementary Material

## Data Availability

All underlying datasets and codes are available upon reasonable request.
