# Supplementary material for: Clean Energy and Health Expenditure in an African Setting: A Socioeconomic Analysis of Household Costs and Willingness to Pay
Source: J Health Econ Outcomes Res. 2026 Mar 20;13(1):93–102. doi: 10.36469/001c.158931 (PMC13006001; doi:10.36469/001c.158931)
Supplement: Online Supplementary Material [file jheor_2026_13_1_158931_336135.pdf]

Journal of Health Economics and Outcomes Research

Online Supplementary Material

Clean Energy and Health Expenditure in an African Setting: A Socioeconomic Analysis of Household Costs and Willingness to Pay. *JHEOR*. 2026;13(1):93-102. doi:10.36469/jheor.2026.158931

This supplementary material has been provided by the authors to give readers additional information about their work.

TABLE OF CONTENTS

APPENDIX 1: Clean Cooking, Household Energy Costs, and Health Expenditures 3

Table S1. Fuel Stacking Patterns by Cooking Group 3

Table S2. Expenditure Outcomes (Descriptive) 4

APPENDIX 2: Two-Part Model (Health Expenditure) 4

APPENDIX 3: Model-Based Cost-Utility Analysis 4

Table S3. Capital Costs Were Annualised Assuming a 5-Year Lifespan 4

Table S4. DALY Estimation (Base Case) 4

Table S5. ICER Scenarios 5

Table S6. Probabilistic Sensitivity Analysis 5

APPENDIX 4: Exploratory Simulation Analysis 5

APPENDIX 5: Analytical Assumptions 6

Cost-Utility Analysis Assumptions 6

Exploratory Simulation Assumptions 7

This is an open-access article distributed under the terms of the Creative Commons Attribution 4.0 International License (CCBY-4.0). View this license's legal deed at http://creativecommons.org/licenses/by/4.0 and legal code at http://creativecommons.org/licenses/by/4.0/legalcode for more information.

APPENDIX 6: FIGURES 8

Figure S1: Boxplot of Monthly Cooking-Energy Expenditure by Cooking Group (Outliers Suppressed) 8

Figure S2: Kernel Density of Monthly Cooking-Energy Expenditure by Cooking Group 8

Figure S3: Boxplot of Annual Health Expenditure (Sum of Reported Treatment Costs) by Cooking Group (Outliers Suppressed) 9

Figure S4: Kernel Density of Annual Health Expenditure by Cooking Group 9

Figure S5: Predicted Probability of Reporting Reduced Healthcare Costs by Cooking Fuel Group (Exploratory Simulation 10

APPENDIX 7: Questionnaire 11

APPENDIX 8: STROBE Statement—Checklist of Items That Should Be Included in Reports of Observational Studies 23

Appendix 1. Clean cooking, household energy costs and health expenditures

Clean-only cooking was defined as households reporting only LPG and/or electricity for cooking (no reported use of firewood, charcoal, kerosene, or straw/dung). Any polluting fuel use (with or without LPG/electricity) was classified as 'Any polluting' to explicitly capture fuel stacking.

Table S1. Fuel stacking patterns by cooking group

Cooking fuels reported	Any polluting (n)	Clean-only (n)
**Liquefied petroleum gas**	0	221
**Liquefied petroleum gas, Electricity**	0	15
**Electricity**	0	3
**Charcoal**	6	0
**Charcoal, Kerosene, Liquefied petroleum gas**	1	0
**Charcoal, Kerosene, Others**	1	0
**Charcoal, Liquefied petroleum gas**	9	0
**Electricity, Others**	1	0
**Firewood**	2	0
**Firewood, Charcoal, Liquefied petroleum gas**	7	0
**Firewood, Charcoal, Liquefied petroleum gas, Electricity**	1	0
**Firewood, Charcoal, Others**	1	0
**Firewood, Liquefied petroleum gas**	8	0
**Kerosene**	1	0
**Kerosene, Liquefied petroleum gas**	4	0

Table S2. Expenditure outcomes (descriptive)

Group	N	Monthly energy mean (₦)	Monthly energy median (₦)	Annual health mean (₦)	Annual health median (₦)
**Any polluting**	53	15611	11000	19785	0
**Clean-only**	239	14140	10000	28087	0

Appendix 2. Two-part model (health expenditure)

Given the heavy right-skew and many zeros in reported treatment spending, we used a two-part model: (i) logistic regression for any annual health spending (>0), and (ii) a gamma GLM with log link for positive spending. Covariates: age, sex, urban/rural, education, household size, and cooking group.

Adjusted mean annual health expenditure was estimated at ₦52,034 for households with any polluting fuel use and ₦31,033 for clean-only households (difference: ₦-21,001). These estimates are descriptive and should not be interpreted causally in a cross-sectional design.

These adjusted estimates reflect cross-sectional associations and should not be interpreted as causal effects of fuel type on healthcare expenditures.

Appendix 3. Model-based cost-utility analysis

To complement the descriptive expenditure comparisons, we conducted a model-based cost-utility analysis using a one-year time horizon and a household perspective.

Cost inputs

Annual household cooking costs were defined as:

$$C_{total} = C_{fuel} + C_{capex}$$

Table S3. Capital costs were annualised assuming a five-year lifespan

Group	Annual cost (₦)	Annual cost (US$)
**Polluting fuels**	184,000	125
**Clean-only fuels**	122,000	83
Increment (clean – polluting)	**−62,000**	**−42**

Currency conversion: ₦1475 per US$1

Table S4. DALY estimation (base case)

Step	Value
**HAP DALYs in Nigeria**	7,000,000
**Population using polluting fuels**	77%

**DALY per exposed person**	0.035
**Mean household size**	4.6
**DALY per polluting household**	0.161
**Adoption effectiveness**	55%
**DALY averted per HH-year**	0.089
**Conservative modelling value**	**0.05**

Employed national household air-pollution burden estimates

Table S5. ICER scenarios

Scenario	DALYs averted	Incremental cost (US$)	ICER	Interpretation
**Conservative**	0.03	−42	Dominant	Cost-saving
**Base case**	0.05	−42	Dominant	Cost-saving
**Optimistic**	0.10	−42	Dominant	Cost-saving

Cost-effectiveness threshold: Nigeria GDP per capita: **US$2160 per DALY averted**

Table S6. Probabilistic sensitivity analysis

Outcome	Value
**Iterations**	10,000
**Probability cost-saving**	0.71
**Probability cost-effective at $2160**	0.97

Appendix 4. Exploratory simulation analysis

Due to the small number of respondents with analysable data on perceived healthcare cost reductions, an exploratory simulation was conducted as a sensitivity exercise. This analysis was not intended to provide empirical evidence or causal estimates, but rather to illustrate potential patterns under hypothetical larger samples. All simulation results should therefore be interpreted as hypothesis-generating only.

A synthetic dataset comprising 300 observations was generated to reflect the key demographic and energy-use patterns observed in the original survey. Specifically, 80% of simulated households were assigned to the clean energy group and 20% to the polluting fuel group, mirroring the empirical distribution of energy use in the study sample (239 clean users and 53 polluting users out of 292 respondents).

A binary outcome variable representing whether the household reported reduced healthcare costs was simulated using illustrative probabilities of 60% for clean energy users and 20% for polluting fuel users. These values were selected to reflect directional patterns observed during preliminary exploration of the original data and were not intended to represent causal effects or validated effect sizes.

Demographic variables were also simulated to represent a plausible cross-section of Nigerian households. Age of the household head was drawn from a normal distribution (mean 45 years,

standard deviation 12 years). Gender was randomly assigned with equal probability, and education level was coded as an ordinal variable ranging from 0 (no formal education) to 3 (tertiary education).

A logistic regression model was then fitted to estimate the association between clean energy use and the likelihood of reporting reduced healthcare costs, adjusting for age, gender, and education. Model coefficients, standard errors, and predicted probabilities were examined to explore group-level patterns under the simulated conditions.

As expected given the constructed probabilities, the simulated analysis showed a higher predicted probability of reporting reduced healthcare costs among clean energy users compared with polluting-fuel users. However, these results are entirely assumption-driven and reflect the parameters specified in the simulation rather than observed causal relationships. They are presented solely to illustrate how larger samples or longitudinal designs might detect differences if such effects were present.

Appendix 5: Analytical assumptions

Cost-utility analysis assumptions

The model-based cost-utility analysis was conducted under the following assumptions:

1. **Time horizon and perspective**
   The analysis was conducted from the household perspective over a one-year time horizon.
2. **Cost components**
   Annual household cooking costs included:
   - Annual fuel expenditures, and
   - Annualised capital costs for stoves and cylinders.
     Capital costs were annualised assuming a five-year lifespan.
3. **Fuel-use scenarios**
   Two scenarios were modelled:
   - Clean-only fuel use (LPG or electricity).
   - Any polluting fuel use (including fuel stacking).
4. **Health effects (DALYs averted)**
   Health benefits were estimated using a burden-share approach based on established household air-pollution attributable burden estimates.
   Three scenarios were modelled:
   - Conservative: 0.03 DALYs averted per household-year
   - Base case: 0.05 DALYs averted per household-year
   - Optimistic: 0.10 DALYs averted per household-year
5. **Currency conversion**
   All costs were converted from Nigerian naira to US dollars using a period-average exchange rate of **₦1,475 per US$1**.
6. **Cost-effectiveness thresholds**
   Cost-effectiveness was assessed against reference willingness-to-pay thresholds of:
   - US$500 per DALY averted
   - US$1,000 per DALY averted
   - Nigeria’s GDP per capita (US$2,160 per DALY averted)
7. **Uncertainty analysis**
   Deterministic scenario analyses and probabilistic sensitivity analysis (10,000 iterations) were conducted to assess uncertainty in cost and effectiveness parameters.

Exploratory simulation assumptions

The exploratory simulation was conducted as a hypothesis-generating sensitivity exercise under the following assumptions:

1. **Distribution of energy use**
   Eighty percent of simulated households were assigned to clean fuels and 20% to polluting fuels, reflecting proportions observed in the study dataset.
2. **Probability of healthcare cost reduction**
   Clean-fuel households were assigned a 60% probability of reduced healthcare costs, compared with 20% among polluting-fuel households.
   These values reflect directional patterns observed in exploratory analyses and do not represent causal estimates.
3. **Age distribution**
   Household heads' ages were simulated from a normal distribution with a mean of 45 years and a standard deviation of 12 years, consistent with the survey sample.
4. **Gender and education**
   - Gender was assigned with equal probability.
   - Education was simulated as a categorical variable ranging from no formal education to tertiary education, with approximately uniform distribution.
5. **Measurement assumptions**
   All variables were assumed to be measured without error.
6. **Model structure**
   The logistic regression model assumed additive and independent effects of predictors on the log-odds of reduced healthcare costs, with no interaction terms.
7. **Sampling assumption**
   The simulated dataset was generated as a random sample from a population with characteristics similar to those observed in the study.

Appendix 6. Figures

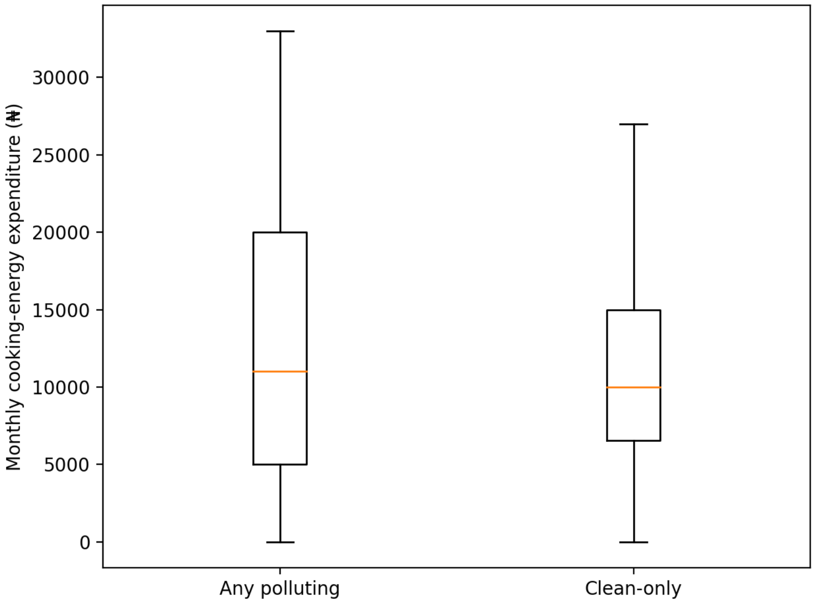

Figure S1. Boxplot of monthly cooking-energy expenditure by cooking group (outliers suppressed).

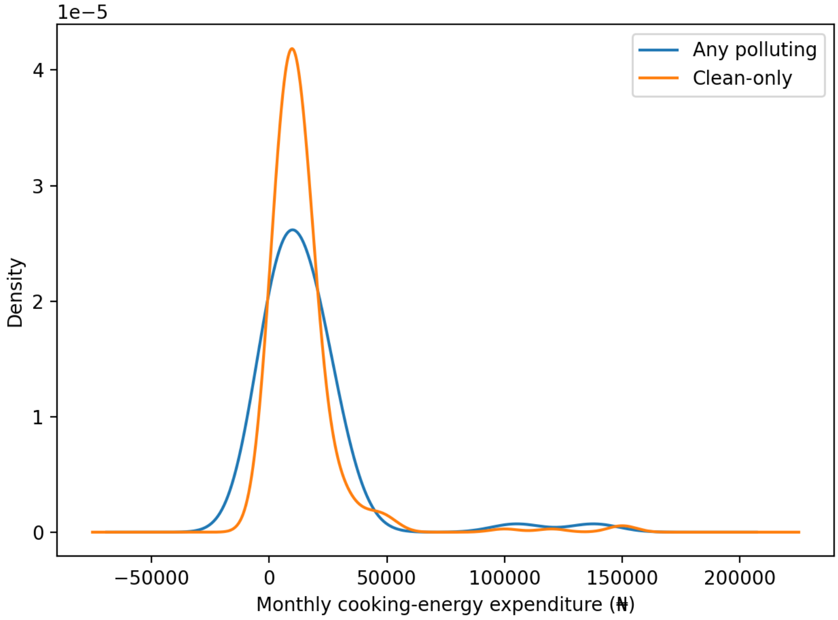

Figure S2. Kernel density of monthly cooking-energy expenditure by cooking group.

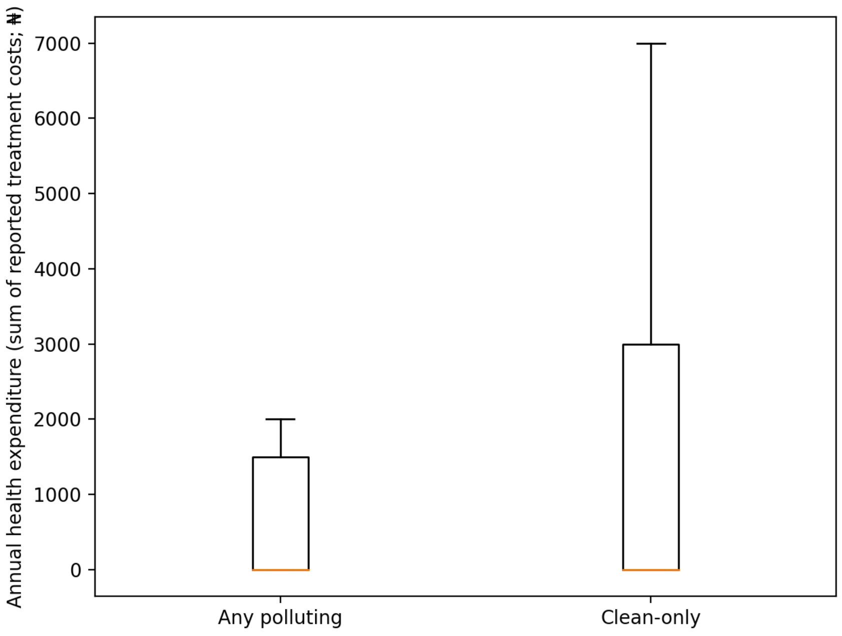

Figure S3. Boxplot of annual health expenditure (sum of reported treatment costs) by cooking group (outliers suppressed).

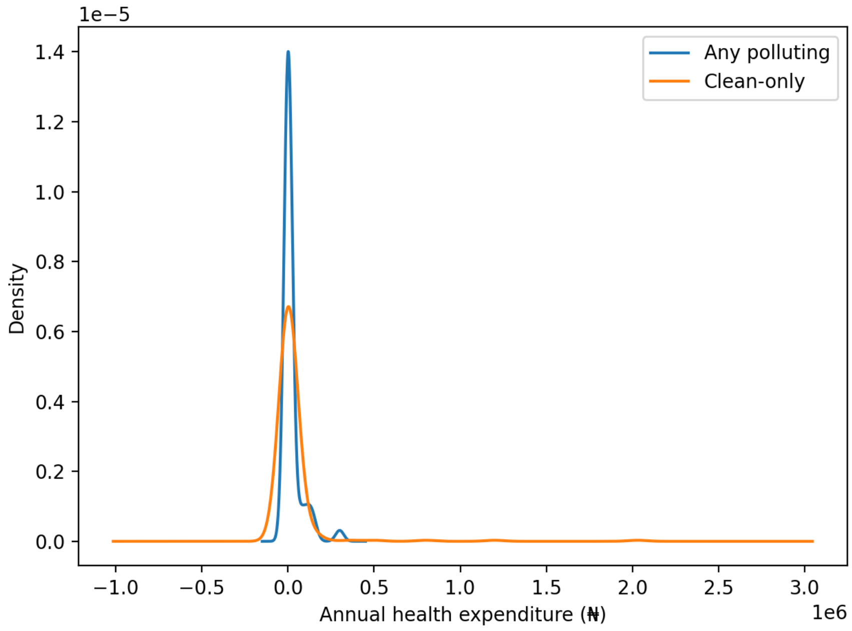

Figure S4. Kernel density of annual health expenditure by cooking group.

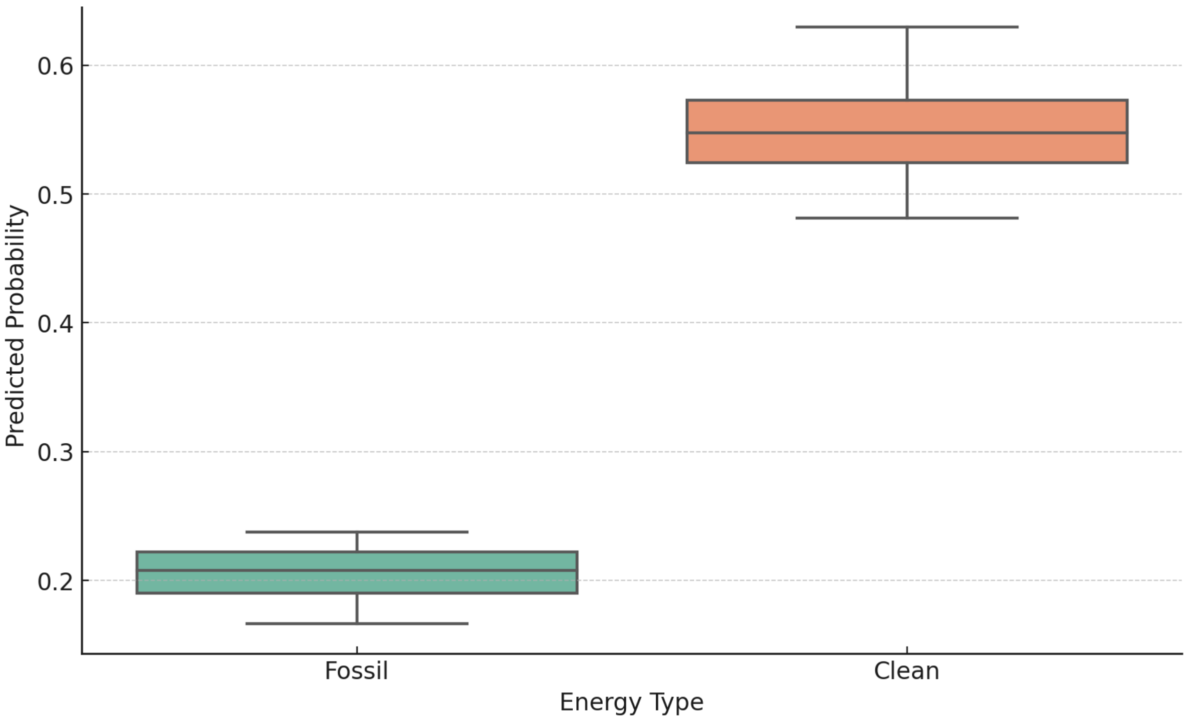

Figure S5. Predicted probability of reporting reduced healthcare costs by cooking fuel group (exploratory simulation).

Predicted probability of reporting reduced healthcare costs by fuel type in the exploratory simulation. Results are assumption-driven and presented for illustrative purposes only.

Appendix 7. Questionnaire

Cost-Benefit Analysis of Cleaner Energy Sources on Household Health

The purpose of this study is to understand the costs and benefits of using cleaner energy sources in households and their impact on health in rural communities. Your participation is voluntary and your responses will be kept confidential and only be used for research purposes. The survey will take approximately 15 minutes. Thank you..

SECTION A: IDENTIFICATION

Date of interview

yyyy-mm-dd hh:mm

Name of Enumerator

Phone number of enumerator

Name of respondent

Phone number of respondent

Geo-political zone

- North East
- North Central
- North West
- South East
- South South
- South West

State

- ◯ Abia
- ◯ Adamawa
- ◯ Akwa Ibom
- ◯ Anambra
- ◯ Bauchi
- ◯ Bayelsa
- ◯ Benue
- ◯ Borno
- ◯ Cross River
- ◯ Delta
- ◯ Ebonyi
- ◯ Edo
- ◯ Ekiti
- ◯ Enugu
- ◯ Gombe
- ◯ Imo
- ◯ Jigawa
- ◯ Kaduna
- ◯ Kano
- ◯ Katsina
- ◯ Kebbi
- ◯ Kogi
- ◯ Kwara
- ◯ Lagos
- ◯ Nasarawa
- ◯ Niger
- ◯ Ogun
- ◯ Ondo
- ◯ Osun
- ◯ Oyo
- ◯ Plateau
- ◯ Rivers
- ◯ Sokoto
- ◯ Taraba
- ◯ Yobe

- Zamfara
- FCT

LGA

Name of community

Record your current location

latitude (x.y °)

longitude (x.y °)

altitude (m)

accuracy (m)

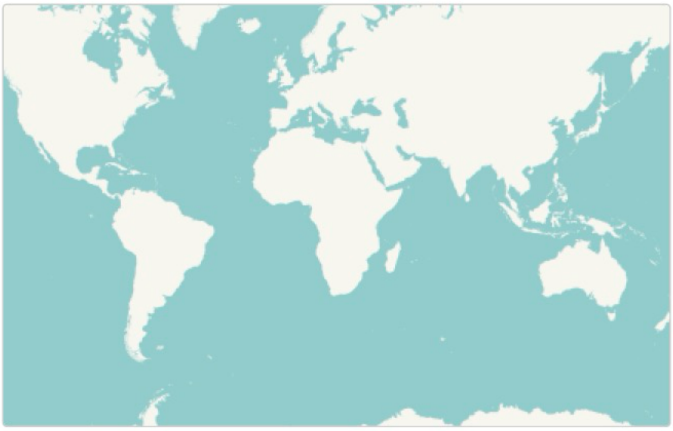

SECTION B: Demographic and socioeconomic characteristics of the respondent

Sex of household head

- Male
- Female

Marital status of the household head

- Single
- Married
- Divorced/Separated
- Widow/Widower

Age of the household head

Spouse Age

Level of education of household head

- ◯ Primary graduate (i.e. First School Leaving Certificate)
- ◯ Some secondary
- ◯ Some Primary
- ◯ College/University graduate
- ◯ None
- ◯ Some college/university
- ◯ Secondary graduate (i.e. O' Level/SSCE)

Level of education of spouse of the household head

- ◯ None
- ◯ Some primary
- ◯ Primary graduate (i.e. First School Leaving Certificate)
- ◯ Some secondary
- ◯ Secondary graduate (i.e. O' Level/SSCE)
- ◯ Some college/university
- ◯ College/University graduate

Location

- ◯ Urban
- ◯ Rural

Households size

____

Number of adults (18+)

____

Number of children (under 18)

____

Main occupation of the household head

____

Household head years of experience

____

Main occupation of spouse

____

Spouse years of experience

____

SECTION C: ENERGY USE AND COST FOR COOKING

Current energy use for cooking

Which of the following energy do you use for cooking (select all that apply)

- ☐ Firewood
- ☐ Charcoal
- ☐ Kerosene
- ☐ Straw or dung
- ☐ Liquid petroleum gas
- ☐ Electricity
- ☐ Others

Others specify

____

How much did you spend on firewood monthly?

____

How much did you spend on charcoal monthly?

____

How much did you spend on kerosene monthly?

____

How much did you spend on straw or dung monthly?

____

How much did you spend on LPG monthly?

____

How much did you spend on Electricity monthly?

____

How much did you spend on other energy source monthly?

____

What was the initial cost of your charcoal stove (s)?

What was the initial cost of your kerosene stove(s)?

What was the initial cost of your Gas cylinder (s)?

What was the initial cost of your Electric cooker(s)?

What was the initial cost of any other cooking source(s)?

Section C2: ENERGY USE AND COST FOR LIGHTING

Current energy use for lighting.

Which of the following energy do you use for lighting (select all that apply)

- [ ] Electricity
- [ ] Solar PV
- [ ] Kerosene
- [ ] Candle
- [ ] Torch (Non-rechargeable)
- [ ] Others

Others specify

How much do you spend on electricity bill per month?

How much do you spend in Solar PV maintenance per year?

How much do you spend on kerosene per month?

How much do you spend on candle per month?

____

How much do you spend on dry cell batteries? (Non-rechargeable batteries) per month?

____

How much do you spend on other lighting energy source per month?

____

What is the initial cost of prepaid meter in your home?

____

What is the initial cost of rechargeable torch, lamps

____

What is the initial cost of Solar PV? (i.e. Installation, Panel, batteries etc.)

____

What is the initial cost of Kerosene lamp(s)

____

What is the initial cost of non-rechargeable torch?

____

What is the initial cost of any other lighting energy source in your home?

____

SECTION D: Health Impacts

Health impact

Has anyone in your household experienced any of the following health issues in the last one year? Select all that apply

- [ ] Cough
- [ ] Bronchitis
- [ ] Pneumonia
- [ ] Eye irritation or infections
- [ ] Skin cancer
- [ ] Skin pigmentation
- [ ] Cardiovascular disease
- [ ] Cataracts
- [ ] Headaches and lung cancer
- [ ] Others
- [ ] Not applicable

Others specify

____

How much was spent in treating cough weekly?

____

How much was spent in treating cough monthly?

____

How much was spent in treating cough yearly?

____

How much was spent in treating Bronchitis weekly?

____

How much was spent in treating Bronchitis monthly?

____

How much was spent in treating Bronchitis yearly?

____

How much was spent in treating Pneumonia weekly?

____

How much was spent in treating Pneumonia monthly?

____

How much was spent in treating Pneumonia yearly?

____

How much was spent in treating Eye irritation or infection weekly?

____

How much was spent in treating Eye irritation or infection monthly?

____

How much was spent in treating Eye irritation or infection yearly?

____

How much was spent in treating Skin cancer weekly?

____

How much was spent in treating Skin cancer monthly?

____

How much was spent in treating Skin cancer yearly?

____

How much was spent in treating Skin pigmentation weekly?

____

How much was spent in treating Skin pigmentation monthly?

____

How much was spent in treating Skin pigmentation yearly?

____

How much was spent in treating Cardiovascular diseases weekly (e.g., stroke, heart attack)

____

How much was spent in treating Cardiovascular diseases monthly (e.g., stroke, heart attack)

____

How much was spent in treating Cardiovascular diseases yearly (e.g., stroke, heart attack)

____

How much as spent in treating Cataracts weekly?

____

How much as spent in treating Cataracts monthly?

____

How much as spent in treating Cataracts yearly?

____

How much was spent in treating Headaches and Lung cancer weekly?

____

How much was spent in treating Headaches and Lung cancer monthly?

____

How much was spent in treating Headaches and Lung cancer yearly?

____

How much did you spend on any other health related disease

____

SECTION E: Willingness to Pay for Cleaner Energy Sources

Are you aware of cleaner energy sources such as solar cookers, improved cookstoves, or biogas?

- ◯ Yes
- ◯ No

Would you be willing to switch to a cleaner energy source if it were available and affordable?

- ◯ Yes
- ◯ No
- ◯ Maybe

What benefits do you expect from using cleaner energy sources?

- [ ] Improved health
- [ ] Reduced energy costs
- [ ] Environmental protection
- [ ] Convenience

How much more or less will you be willing to pay per month for a cleaner energy source that improves your household's health relative to the current expenditure?

- ○ Less than the current expenditure
- ○ Same as the current expenditure
- ○ Between1-20% more than current expenditure
- ○ Between 21-50% more than current expenditure
- ○ More than 50% of current expenditure

How much would you be willing to pay per month for Biomass energy (animal and plant waste)?

____

How much would you be willing to pay per month for Solar energy (solar PV)?

____

How much would you be willing to pay per month for Wind energy?

____

How much would you be willing to pay per month for Small hydropower?

____

Do you use Biomass energy (animal and plant waste)

- ○ Yes
- ○ No

What is the expected number of years or life span of the biomass energy (animal and plant waste)?

____

Do you use Solar energy (Solar PV)

- ○ Yes
- ○ No

What is the expected number of years or life span of the Solar energy?

____

Do you use Wind energy?

- ◯ Yes
- ◯ No

What is the expected number of years or life span of the Wind energy?

____

DO you use Small hydropower?

- ◯ Yes
- ◯ No

What is the expected number of years or life span of the Small hydropower?

____

Since you started using clean energy, has the cost of healthcare services reduced?

- ◯ Yes
- ◯ No
- ◯ Not sure
- ◯ Not applicable

Give an estimate on the reduction of healthcare services (drugs) and consultation per year

____

What barriers do you think might prevent you from adopting cleaner energy sources?
Select all that apply

- ☐ High initial cost
- ☐ Lack of availability
- ☐ Lack of information
- ☐ Cultural preferences
- ☐ Others

Others specify 1:

____

Others specify 2:

____

Thank you for your time and participation

Appendix 8. STROBE Statement—checklist of items that should be included in reports of observational studies

	Item No.	Recommendation	Page No.	Relevant text from manuscript
**Title and abstract**	1	(*a*) Indicate the study's design with a commonly used term in the title or the abstract	Title page, 1	
		(*b*) Provide in the abstract an informative and balanced summary of what was done and what was found	1	
**Introduction**				
Background/rationale	2	Explain the scientific background and rationale for the investigation being reported	2	
Objectives	3	State specific objectives, including any prespecified hypotheses	3	
**Methods**				
Study design	4	Present key elements of study design early in the paper	3	
Setting	5	Describe the setting, locations, and relevant dates, including periods of recruitment, exposure, follow-up, and data collection	3	
Participants	6	(*a*) *Cohort study*—Give the eligibility criteria, and the sources and methods of selection of participants. Describe methods of follow-up<br>*Case-control study*—Give the eligibility criteria, and the sources and methods of case ascertainment and control selection. Give the rationale for the choice of cases and controls<br>*Cross-sectional study*—Give the eligibility criteria, and the sources and methods of selection of participants	3-5	

		(*b*) *Cohort study*—For matched studies, give matching criteria and number of exposed and unexposed<br><br>*Case-control study*—For matched studies, give matching criteria and the number of controls per case	N/A
Variables	7	Clearly define all outcomes, exposures, predictors, potential confounders, and effect modifiers. Give diagnostic criteria, if applicable	4-5
Data sources/ measurement	8*	For each variable of interest, give sources of data and details of methods of assessment (measurement). Describe comparability of assessment methods if there is more than one group	*5-6*
Bias	9	Describe any efforts to address potential sources of bias	6
Study size	10	Explain how the study size was arrived at	6

Quantitative variables	11	Explain how quantitative variables were handled in the analyses. If applicable, describe which groupings were chosen and why	6-7
Statistical methods	12	(*a*) Describe all statistical methods, including those used to control for confounding	6-7
		(*b*) Describe any methods used to examine subgroups and interactions	7
		(*c*) Explain how missing data were addressed	7
		(*d*) *Cohort study*—If applicable, explain how loss to follow-up was addressed<br><br>*Case-control study*—If applicable, explain how matching of cases and controls was addressed<br><br>*Cross-sectional study*—If applicable, describe analytical methods taking account of sampling strategy	5-6
		(*e*) Describe any sensitivity analyses	7

**Results**			
Participants	13*	(a) Report numbers of individuals at each stage of study—eg numbers potentially eligible, examined for eligibility, confirmed eligible, included in the study, completing follow-up, and analysed	8
		(b) Give reasons for non-participation at each stage	8
		(c) Consider use of a flow diagram	N/A
Descriptive data	14*	(a) Give characteristics of study participants (eg demographic, clinical, social) and information on exposures and potential confounders	8
		(b) Indicate number of participants with missing data for each variable of interest	8
		(c) *Cohort study*—Summarise follow-up time (eg, average and total amount)	N/A
Outcome data	15*	*Cohort study*—Report numbers of outcome events or summary measures over time	N/A
		Case-control study—Report numbers in each exposure category, or summary measures of exposure	N/A
		Cross-sectional study—Report numbers of outcome events or summary measures	*9*
Main results	16	(*a*) Give unadjusted estimates and, if applicable, confounder-adjusted estimates and their precision (eg, 95% confidence interval). Make clear which confounders were adjusted for and why they were included	9
		(*b*) Report category boundaries when continuous variables were categorized	9
		(*c*) If relevant, consider translating estimates of relative risk into absolute risk for a meaningful time period	N/A

Other analyses	17	Report other analyses done—eg analyses of subgroups and interactions, and sensitivity analyses	9

**Discussion**			
Key results	18	Summarise key results with reference to study objectives	10
Limitations	19	Discuss limitations of the study, taking into account sources of potential bias or imprecision. Discuss both direction and magnitude of any potential bias	11
Interpretation	20	Give a cautious overall interpretation of results considering objectives, limitations, multiplicity of analyses, results from similar studies, and other relevant evidence	11-12
Generalisability	21	Discuss the generalisability (external validity) of the study results	11-12
**Other information**			
Funding	22	Give the source of funding and the role of the funders for the present study and, if applicable, for the original study on which the present article is based	13

*Give information separately for cases and controls in case-control studies and, if applicable, for exposed and unexposed groups in cohort and cross-sectional studies.

Note: An Explanation and Elaboration article discusses each checklist item and gives methodological background and published examples of transparent reporting. The STROBE checklist is best used in conjunction with this article (freely available on the Web sites of PLoS Medicine at http://www.plosmedicine.org/, Annals of Internal Medicine at http://www.annals.org/, and Epidemiology at http://www.epidem.com/). Information on the STROBE Initiative is available at www.strobe-statement.org.
